# Nettle (*Urtica dioica*) Additive as a Growth Promoter and Immune Stimulator in Fish

**DOI:** 10.1155/2023/8261473

**Published:** 2023-02-21

**Authors:** Mahyar Zare, Noah Esmaeili, Simona Paolacci, Vlastimil Stejskal

**Affiliations:** ^1^Faculty of Fisheries and Protection of Waters, South Bohemian Research Center of Aquaculture and Biodiversity of Hydrocenoses, Institute of Aquaculture and Protection of Waters, University of South Bohemia in Ceske Budejovice, Husova Tř. 458/102, 370 05 České Budějovice, Czech Republic; ^2^Institute for Marine and Antarctic Studies, University of Tasmania, Hobart, Australia; ^3^Bantry Marine Research Station, Gearhies, Bantry, Co. Cork P75 AX07, Ireland

## Abstract

Aquaculture will become an important food production sector for humans in the coming decades. However, disease outbreaks can be considered a significant obstacle to continually developing aquaculture. Plant powders and extracts are natural feed additives that, due to their bioactive compounds, including phenolic compounds, proteins, vitamins, and minerals, have antistress, antiviral, antibacterial, and antifungal effects on fish. One of these herbs is nettle (*Urtica dioica*), which has a long history of being used in traditional medicine. While it has been widely investigated in mammalian medicine, few studies have been done on aquaculture species. The positive effect of this herb on the growth performance, hematology, blood biochemistry, and immune system of fish species has been observed. When fish were exposed to pathogens, nettle-fed fish showed a higher survival rate and less stress than controls. Therefore, this literature review is aimed at reviewing the use of this herb in fish diets and its impacts on growth performance, hematology, blood biochemistry, liver enzymes, immune system stimulation, and challenges with pathogens.

## 1. Introduction

Aquaculture plays a vital role in supplying protein-rich food to humans [1]. Finfish and crustacean production occurs in different environments, from inland aquaculture to marine aquaculture, with annual production of more than 82 million tons in 2018. The aquaculture production demands hormones, antibiotics, and growth promoters to secure high and aquatic sustainable production [2]. Antibiotics are consumed widely in aquaculture, and in a recent report, at least 67 different kinds of antibiotics were used in 2008 and 2018 [3]. The use of antibiotics and other chemical substances in aquaculture raises concerns about food safety and quality, public health, the environment, and fish and human welfare [4]. These substrates cause pathogen resistance to antibiotics, and their accumulation in aquatic species is harmful when humans consume them [5]. Therefore, researchers and industry are seeking sustainable alternative options. Among the different options, herbal additives are sustainable and green alternatives to hormones and antibiotics used in fish feeds [6] to improve growth and health. Bioactive compounds extracted from herbs such as alkaloids, tannins, flavonoids, terpenoids, glycosides, and phenols [7] are responsible for positive outcomes, such as improved growth and health in fish [6]. Thus, in the last decades, several kinds of literature have reviewed the effects of different plant additives on aquatic species feeds [6, 8, 9] as well as livestock animals [10, 11].

Nettle (*Urtica dioica*) has been used for centuries among different medicinal herbs. Nettle is one of the nine plants invoked in the pagan Anglo-Saxon Nine Herbs Charm, recorded in 10^th^-century traditional medicine. This herb has a long list of features and benefits for humans [12]. For example, it has been known as a natural antihistamine diuretic, arthritis, anemia, hay fever blood purifier, astringent, antioxidant, antimicrobial, and anti-inflammatory agent [13]. Nettle is frequently used for allergies, anemia, a sluggish thyroid, restoring adrenal tonics, arthritis, and rheumatism [14]. The first reports about the use of nettle in the livestock diet are related to poultry in 1936 [15] and pig in 1974 [16]. Nettle supplements can result in a positive effect on growth, feed utilisation, blood lipids, antioxidants, and the immune system of poultry nutrition [17]. Administration of 1% nettle powder into broiler feed improved weight gain (WG), FCR, and liver antioxidant enzyme, including superoxide dismutase (SOD) and catalase (CAT), in the liver after 42 days of feeding trial [18]. In contrast, nettle powder and extract at levels 0.5 and 1% in broiler feed did not significantly improve WG, FCR, feed intake, blood serum cholesterol, triglyceride, glucose, alanine aminotransferase (ALT), and alkaline phosphatase (ALP) [19]. Studies related to supplementing nettle in the diets of pigs are limited. In the only available research, the use of 0.05 and 0.1% of nettle water extract in diets of pigs significantly improved fat and protein deposition and n-3 omega 3, decreased blood serum high-density lipoproteins, and had no change in cholesterol compared to the control [20]. But fishes live in different environments, temperatures, and salinity and are carnivorous, omnivorous, and herbivorous with a different physiology of digestion. Due to the long list of benefits, researchers in aquaculture have tested this herb in fish diets. Based on the literature, Awad and Austin [21] first tried 1% nettle in rainbow trout (*Oncorhynchus mykiss*). The results showed significant improvement in the immune system, including respiratory burst, lysozyme enzyme activity, bactericidal activity, and a high survival rate (96%), compared to the control (32%) after a 10-day challenge with *Aeromonas hydrophila*. In addition, 1% nettle increased hematocrit (Ht%) compared to the control. Nettle, as a medicinal plant additive, has been evaluated in fish feeds and has improved growth performance, proximate body composition [22], immune system, and resistance against pathogens [23, 24] and preserved fish fillet quality [25]. The literature has also briefly reviewed the effect of nettle on fish growth performance and the immune system [26]. There is a gap in knowledge regarding the effect of nettle on other parameters, such as proximate body composition, hematology, serum biochemistry, liver enzymes, immune stimulant, and resistance against pathogens. Moreover, we included the latest nettle articles in the present review. Therefore, this review paper is aimed at providing a comprehensive and updated view of the effect of nettle on aquaculture species and a short view of animals' monogastric farms (poultry and pig) to be compared with fish.

## 2. Botanical Characteristics and Bioactive Compounds of Nettle

Nettle, common nettle, or stinging nettle, which are all synonyms, is a perennial plant that belongs to the family of *Urticaceae*. It is found in moderate regions of Europe, Asia, North Africa, and North America up to 1,800 m altitude [27]. It is 2 to 4 meters high and creates sharp leaves [13], and both sides of the leaves are covered with tiny trichomes [27]. Moreover, it has white to yellowish flowers [13] and reddish or yellowish stems. A wide range of compounds was isolated from aerial parts, leaves, stems, flowers, seeds, and roots of nettle [27]. Essential amino acids alanine (6.4 *μ*g/g), 4-aminobutyrate (GABA, 3.4 *μ*g/g), glutamic acid (6.0 *μ*g/g), isoleucine (1.9 *μ*g/g), leucine (7.6 *μ*g/g), phenylalanine (2.4 *μ*g/g), proline (5.7 *μ*g/g), tyrosine (1.2 *μ*g/g), and valine (3.0 *μ*g/g) are available in this herb. Organic acids, including malic acid (12.8 *μ*g/g), acetic acid (29.3 *μ*g/g), citric acid (3.6 *μ*g/g), succinic acid (2.6 *μ*g/g), and formic acid (6.9 *μ*g/g), were found in the leaves. Some lipids, including steroids and triterpenoids 3-b-sitosterol (40 mg/kg), sitosterol-b-D-glucoside (30 mg/kg), (60-O-palmitoyl)-sitosterol-3-O-b-D-glucoside (5.6 mg/kg), 24R-ethyl-5a-cholestane3b,6a-diol (3.3 mg/kg), 7b-hydroxy-sitosterol (2.4 mg/kg), 7a-hydroxy sitosterol (2.9 mg/kg), 7b-hydroxy-sitosterol-b-D-glucoside (2.7 mg/kg), and 7a-hydroxy-sitosterol-b-D-glucoside (2.0 mg/kg), were detected in roots. Fatty acids, terpenoids, phenolic compounds, and volatile compounds were discovered in leaves, roots, seeds, and stems [27]. Furthermore, the nettle supplement (powder/extract) contains a wide range of vitamins and minerals, including both fat-soluble vitamins A, D, E, and K and water-soluble vitamins, such as vitamin C and the B vitamins (B1, B2, B3, and B9) (Supplementary table 1) [28].

In contrast, some antinutritional compounds, such as phytates (phytic acid), oxalates, and saponins, were found in nettle, limiting their supplementation level in diets and negatively impacting fish growth and health. These compound levels depend on the nettle's harvesting location and growth stage [29]. Therefore, the optimum dosage is required to positively affect growth and other physiological parameters; however, it has a certain level of antinutritional effects. These antinutritional effects cause many problems in fish and other animals at excessive levels. For example, phytic acid inhibits the absorption of minerals, such as iron, calcium, manganese, and zinc, in the digestive system of animals [30]. Oxalates can bind with calcium or magnesium in feed to shape insoluble calcium or magnesium oxalate. Then, these salts sediment in the kidneys of animals [31]. Saponins are steroid or triterpenoid glycosides and have shown positive effects, including membrane permeabilizing, immunostimulant, and anticarcinogenic effects in animals. By contrast, excessive levels of saponins demonstrated adverse effects, such as hypocholesterolemia, hypoglycemia, damaged digestion of protein, reduced uptake of vitamins and minerals in the gut, and negative impact on growth, feed intake, and reproduction in humans and animals [32]. Measuring these antinutritional parameters in diets supplemented with nettle can help formulate an optimum diet for fish. These compounds in any herb may react differently, and more research on the antinutritional parameters of nettle is required.

## 3. Preparation of Nettle Powder and Extract

Nettle, like other herbs, has been used in fish diets in two forms: powder and extract. Nettle powder is prepared from aerial parts [33, 34], leaves [22], and stems [35] similar to other herbs. Collected nettle samples were washed with distilled water [24]. Then, the samples were dried in the shade at room temperature [22, 36] and/or in the shade under ambient conditions under a flow of dry air [37]. The benefits of using powdered herbs like nettle include the fact that it is acceptable for fish, is widely available and cheap, and can easily be added to diets. However, sometimes, adding nettle to diets in its powdered form can be difficult to digest for carnivorous species. The antinutritional parameters are sometimes related to fiber, and using powders can cause them to be present in diets more than in the extract. On the other hand, with extracting, we exclude some antinutritional parameters and fiber, making them easier to digest for all fishes. Another benefit of extraction is that only a small amount of nettle is required for diets (usually less than 500 mg/kg). Based on our previous experience, to provide 1 g of dried extract, 200–250 g of powder is required. Therefore, more bioactive compounds can reach fish bodies by formulating diets with the extract. However, the extraction process requires laboratory equipment and is costly. These issues can be generalized to all herbs. Nettle extract, like other herbs, is sourced from aerial parts [38] and roots [39]. In the ethanol method, nettle samples are well-dried in a dark room and ground into a fine powder using a grinder. The prepared powder is then mixed in a 1 L volumetric flask at a ratio of 1 : 5 (*w*/*v*) with 80% ethanol for 48 h using a shaker. Then, the mixture is filtered using a Büchner funnel and filter paper. The primary extract is distilled via rotary distillation at 80°C for 4 h [33]. In the methanol method, after drying under the shade and grinding into good powder, a 50 g sample is percolated with 1 L methanol (40%) for 3 days and then filtered. The solvent is evaporated, and finally, the concentrate is dissolved in 50 mL of deionized water at 50°C [40]. In the acetone method, the nettle sample is dried for 7 days in the shade. The dried sample is ground into a fine powder. The sample is maintained in flasks with acetone (1 : 10 *w*/*v*). Subsequently, the extract is filtered through Whatman No. 1 filter paper, and the extract is concentrated under low pressure at 45°C with the pressure of a vacuum evaporator [38]. Depending on the extraction method and drying temperature, the final nutritional composition of herbs can differ slightly. A comprehensive investigation is required to determine how other extraction methods can affect the nutritional compositions of nettle species. Furthermore, it is interesting to see how fish growth and health can be affected by extraction methods. According to the documented results, both nettle powder (ranging from 0.5% to 12%) [21, 22, 35, 37] and extract with different methods (ranging from 0.01% to 3%) [23, 33, 40] in fish diets demonstrated positive effects on fish growth performance and health status [26]. This shows that both powder and extract of herbs could be effective and that extraction did not add any positive effects. However, their level in diet also matters. Herb powder or extract of nettle, like other herbal medicines, can be dried at room temperature and can be stored in the fridge, preferably at +4°C, until used.

## 4. Effects of Nettle on Growth Performance and Proximate Composition of Fish

Growth performance is the most crucial trait in aquaculture. In normal situations and supposing the same FCR, a system that can grow fish more effectively can be more sustainable and profitable. Farming fish with improved feed efficiency is necessary to reduce production costs and achieve sustainability in the aquaculture industry [41]. Therefore, considering that both phenotypes (growth and phenotype) are crucial for aquaculture, a balance between growth and feed efficiency should be considered. This makes all the more sense when we study the effect of supplements on fish diets, and only increased growth or feed efficiency is insufficient. Nettle is one of these supplements, and in the related literature, both growth and feed efficiency have been discussed. There can be a considerable difference between fish being fed with apparent satiation or limited feeding. The preferable approach is apparent satiation to test palatability when herbs such as nettle are added to diets. The experimental period and size of fish are also other important parameters that can directly affect growth and feed efficiency and should be considered in nettle and other herb studies.

Rainbow trout is the most common cold-water species that has been studied in supplementing herbal medicines to fish diets. This is because rainbow trout is one of the most commonly farmed aquaculture species in Iran and Turkey, where studies on the effects of herbs such as nettle on aquaculture are prevalent [16, 18, 33]. Administration of 3% nettle leaf powder to fish diet (7 g) significantly increased WG and specific growth rate (SGR) after 8 weeks of feeding based on apparent satiation with no change in FCR [22]. This herb significantly improved whole-body proximate protein and ash in this study and decreased fat content. Supplementation of 0.5% nettle powder showed the same result, as FCR was improved as well, in rainbow trout (12 g) after 4 [37] and 8 [24] weeks when fish were fed 3% of body weight. In another study, Awad et al. [42] administered 1% and 2% nettle leaf powder in rainbow trout diets (18 g) and saw an increase in WG and SGR and no change in FCR after an 8-week feeding trial; however, no significant effect on proximate body composition was observed. Studies on extracted nettle revealed the same trend and recorded improved WG, SGR, and FCR. For example, supplementing 3% nettle sourced from aerial parts with ethanol extract in fish (42 g) after 8 weeks of feeding based on 3% of body weight [33] and nettle methanol extract at 0.01% and 0.05% for 4 weeks based on apparent satiation [40] resulted in this output.

Studies that have investigated the effect of herbal supplements on warm-water species are usually found in freshwater. Iran, India, Egypt, and China are the top countries that focus on the effect of herbs, such as nettle, in warm-water species. As most of the studied fish species in this category are omnivorous, the inclusion of high levels of plant-based ingredients, such as nettle, did not impair fish growth. Nettle powder (5% of diet) improved WG, SGR, and FCR in Victoria Labeo (*Labeo victorianus*: 25 g) after 16 weeks of feeding based on 3-4% body weight [36]. In contrast, when beluga (*Huso huso*: 30 g) was fed with a diet including 1% nettle powder, it did not show a significant effect on growth performance after a 60-day feeding trial based on apparent satiation [34]. Similarly, administration of nettle acetone extract (0.015% of diet) after 3 months of feeding based on apparent satiation did not increase growth performance in electric yellow cichlid (*Labidochromis caeruleus*: 0.6 g) [38]. Use of 0.01% and 0.05% methanolic extract of nettle in female convict cichlid (1.3 g) (*Amatitlania nigrofasciata*) diet for 56 days significantly improved WG, SGR, feed intake, and FCR compared to the control [43].

It should be noted that the duration of administration is an essential factor. For example, a 4-week experiment is not enough to draw a solid conclusion. The fish's response to diet can be changed with adaptation (feeding more after 4 weeks or even less feeding due to a lack of palatability). The size of the fish was quite similar in these studies. The reason for these improvements in the abovementioned studies can be biocomponents such as flavonoids (kaempferol, quercetin, and quercitrin) [14], which can increase feed palatability [33, 42] and decrease FCR significantly [24, 37]. Moreover, Awad et al. [42] showed that the administration of 2% nettle powder in a fish diet after a two-month feeding trial could stimulate the stomach enzyme pepsin and other protease activities. Pepsin is an acidic protease found in gastric juice and is a main digestive enzyme that helps feed protein digestion by breaking down proteins into smaller peptides [44]. There is a long list of herbs that improve digestive enzymes and, eventually, growth [45, 46], which is not within the scope of this article. Nettle contains essential amino acids [47], and phenolic compounds, such as vanillic acid (which is used as a flavoring agent), could increase feed palatability [27]. The improved feed intake and digestibility in both cold-water and warm-water species can be explained by these reasons. Interestingly, an increased hemoglobin (Hb) level in experimental groups fed with a nettle supplement could improve growth performance in rainbow trout [22, 24, 37]. Hb, which has a vital function in transporting oxygen [48], affects the metabolism and growth performance of fish [49]. This protein plays a key role in cold-water species, which require more oxygen. The mechanism by which nettle improves Hb is an interesting topic for future studies. In short, different mechanisms can improve growth and feed efficiency by adding nettle to fish diets. For example, its bioactive compounds [27]; ability to modify colonization of various bacteria species in fish gut [50] and improve immunity [22, 40]; antioxidant activities [35]; hematological parameters [22, 33, 37]; blood metabolites [36, 43]; improved lipid, carbohydrate, and protein metabolism [22]; and stimulating digestive enzymes [42] are among them. In most cases, herbs such as nettle cannot change body composition, as the protein and lipid contents of experimental diets are similar. The effects of nettle supplementation on growth performance and proximate body composition of fish species are depicted in Table 1.

## 5. Effects of Nettle on Hematological and Blood Biochemical Parameters in Fish

Hematological parameters, including red blood cells (RBCs), white blood cells (WBCs) count, Hb, and Ht indices, have been known as bioindicators of fish health. Measuring hematological parameters is cheap and assessable and, therefore, commonly measured. The RBC of an organism is responsible for the carrying capacity of dissolved oxygen [51]. WBC is the first line of defense against pathogens [52]. The percentage of Ht shows the volume percentage of RBCs in the blood [53]. Hb is included in RBCs, and it is a globular protein with a quaternary structure constituted of four globin chains (two alpha and two betas) and a prosthetic group called heme bound to each one [54]. Given the diverse critical roles of blood, measuring blood parameters may provide a reliable picture of fish metabolism and health status [55]. As nettle can affect the growth and immunity of fish, it can directly impact hematological parameters. However, this question is raised whether the improved hematological parameter is the reason or/and result of improved growth and immunity.

Collecting blood from rainbow trout is easy, even in fish with a size less than 50 g. Therefore, we can see that hematological parameters have commonly been measured in rainbow trout studies. Nettle has positively affected hematological parameters in this fish species. Administration of 1% and 1.5% of nettle leaf powder in rainbow trout (10-11 g) diet increased WBCs, RBCs, Ht, and Hb after 4- [37] and 8-week [24] feeding trials. Mehrabi et al. [24] reported increased lymphocytes, decreased monocytes, and no significant effects on neutrophil percent by adding nettle to diets. By contrast, supplementing 3% nettle leaf powder in rainbow trout (7 g) diet for 8 weeks increased WBC, neutrophil, and monocyte counts but reduced RBC, Ht, and Hb levels [22]. In another report, Awad and Austin [21] mentioned that 1% nettle leaf powder in rainbow trout diet with an initial average body weight of 15 g after 14 days of feeding did not improve RBC, WBC, Hb, monocyte, and neutrophil counts but increased Ht significantly. It can be hypothesized that 14 days was not enough to change these parameters in rainbow trout. Administration of nettle sourced from aerial parts with ethanol extract in the diet of this species (42 g) at levels of 1%, 2%, and 3% after 4 weeks of feeding did not increase RBCs, WBC count, and monocyte percent but, at a dietary level of 3%, improved Ht, Hb, lymphocyte, and neutrophil percent [33]. However, after an 8-week feeding trial, nettle in this study improved RBC, WBC, Ht, Hb, and neutrophil percent [33]. This is further evidence that the experimental period plays a key role in making changes to blood parameters.

Warm-water species that were experimented with nettle supplementation usually have less blood, and collecting blood from them is a bit harder than from cold-water ones. For example, carps, acipenser, Victoria Labeo, and cichlids belong to this category. Supplementation of 1%, 2%, and 5% nettle leaf powder in Victoria Labeo (25 g) after 4 and 16 weeks in a feeding trial showed significant improvement of RBC, WBC, Ht, and Hb compared to controls [36]. In another study [35], nettle leaf powder (12% of diet) in beluga (204 g) after 4 weeks improved RBC, Ht, and Hb, but WBC, lymphocyte, and eosinophil levels did not show a significant difference. By contrast, in this research, after 8 weeks of the feeding trial, WBC, RBC, Ht, and Hb increased significantly in the nettle group, but not lymphocyte and eosinophil. These results indicate that, again, the time of administration plays a key role. Nobahar et al. [34] studied the effect of 1% nettle powder on beluga, with an initial average body weight of 30 g. In that study, nettle did not increase WBC, RBC, Ht, or Hb after the 60-day feeding trial. While no significant difference was observed in lymphocytes, neutrophils, and monocytes, eosinophils decreased [34]. Blood is responsible for transferring different components, including water, gases, hormones, minerals, immune constituents, nutrients, microorganisms, toxins, and waste products in fish [56]. Thus, the normal range of hematologic parameters shows the fish health status [8]. Furthermore, increased hematological parameters in most cases are a good sign (not in the case of severe infections). Esmaeili [55] reviewed changes in hematological parameters in growth studies (based on more than 400 monitored cases). In most cases, fish with elevated hematologic parameters were healthier and had a higher growth rate. Furthermore, hematological parameters do not show the same trend across treatments and studies. Therefore, a new introduced formula (blood performance) can be a better indicator [55]. To test “blood performance” in nettle studies, we calculated this formula. While many variations in the trend of these parameters were observed, “blood performance,” interestingly, in most of the nettle groups was significantly higher than controls [21, 24, 33, 35, 37]. It can be concluded that the higher hematological parameters in the studied supplemented nettle can be a good sign, as they had higher growth than the controls as well. Higher survival rates and fish resistance to pathogens [24, 33] are more evidence of this claim. Dügenci et al. [57] reported that vitamin C increased iron uptake in fish intestines. Since nettle is rich in vitamin C and iron [47], an increase in RBCs, Ht, and Hb can be attributed to these vitamins' synergistic effect via stimulation of the head kidney [22, 33]. The results of the administration of nettle in the fish diet on hematological parameters are presented in Table 2.

Blood biochemistry reveals the metabolic and health status of fish. They have become powerful tools and increasingly common measures in aquaculture studies. Using plant powder and/or extracts like nettle in the fish diet can improve blood biochemical parameters and enhance fish physiology and health conditions [22, 36, 37]. Nettle leaf powder at levels 3% [22] and 0.5, 1, and 1.5% [24] in rainbow trout (7-10 g) diet improved blood serum total protein and albumin after an 8-week feeding trial [37]. Furthermore, the use of 1% nettle extract (quercetin) in a rainbow trout diet (18 g) increased blood serum total protein after 14 days of feeding [23]. Conversely, 1% nettle leaf powder in a rainbow trout diet (15 g) after 2 weeks of feeding did not significantly affect blood serum total protein [21]. Adel et al. [33] reported that the ethanol extract of nettle aerial parts in rainbow trout (42 g) increased blood serum total protein and decreased blood serum glucose and triglyceride levels after 8 weeks of feeding. Furthermore, using 0.01% and 0.05% methanolic extract of nettle in female convict cichlid diet decreased glucose, triglyceride, and cholesterol. It significantly increased the total protein and albumin levels compared to the control [43]. Supplementation of nettle leaf powder at levels 1, 2, and 5% in Victoria Labeo (25 g) after a 4- and 16-week feeding trial, increased blood serum total protein, albumin, triglyceride, cholesterol, and decreased level of blood glucose [37]. An increase in serum total protein decreased blood serum triglycerides and cholesterol, and no change in glucose levels in beluga (204 g) after 8 weeks was observed [35].

Blood serum total protein and albumin are important factors for vertebrate health [58] and can boost the immune response [59]. It can be concluded that the use of nettle supplements in the fish diet can increase blood serum total protein due to bioactive nettle compounds, such as quercetin [23]. Compared to other herbs, nettle is reasonably high in quercetin (2 mg/100 g), and it is a rich source of antioxidants and vitamins A and C. Increasing blood serum albumin due to nettle supplementation can improve transportation of substances, including bilirubin, metals, ions, enzymes, amino acids, hormones, free fatty acids, drugs, and phospholipids, by providing many surface-charged groups and many particular binding places [22, 24, 37, 60]. There is no study related to the effect of nettle on glucose, cholesterol, and triglyceride in rainbow trout to check the glucose- and lipid-lowering effects of this herb. The glucose-lowering effect of nettle was reviewed in mammal studies [61]. Furthermore, many studies have reported the lipid-lowering effect of this herb in a mammal, which reduces cholesterol, low-density lipoproteins, and triglyceride in blood and improves high-density lipoproteins [62]. Parham et al. [63] reported that nettle stimulated the secretion of insulin and affected the peroxisome proliferator-activated receptor- (PPAR-) gamma agonist and the repressing effect on alpha-glucosidase, which has an inhibitory role in reducing blood glucose levels [64]. Alpha-glucosidase is a carbohydrate-hydrolyzing enzyme such as *α*-amylase and *α*-glucosidase, which is located in the brush border of the small intestine and breaks down oligosaccharides and disaccharides into monosaccharides that are suitable for absorption [65]. Moreover, hydroalcoholic extract of nettle showed a protective and hypoglycemic effect on pancreatic beta cells in hyperglycemic rats, which can be related to antioxidant possessions of nettle [66] such as phenolic compounds including quercetin and kaempferol [27, 67]. Moreover, the efficiency of nettle on fish glucose levels can be related to fish species, parts of nettle used as a supplement, and the type of nettle supplement (powder and/or extract) [35, 36, 67]. Decreasing the level of cholesterol and triglycerides in the blood serum can be related to nettle sterols, including stigmasterol, campesterol, *β*-sitosterol [68], and phenolic compounds [27], which affect cholesterol biosynthesis [69]. In conclusion, the positive effect of nettle on protein, glucose, and lipid metabolism was also observed, like many other herbs. The effects of supplementation nettle in the fish diet on blood biochemical parameters are displayed in Table 3. In the studies where the same output was not observed, there were short-term (2 weeks) studies. This can indicate the importance of the administration period, and at least 4 weeks are required. By matching these data with growth, most of the treatments with higher growth had higher total protein (which can indicate better health). Fish growth is closely related to its health status. A fish with a higher growth rate is more likely to be healthy [55]. Furthermore, improved blood biochemistry due to nettle supplementation coincided with a higher survival rate resistance against stress [33, 36].

## 6. Effects of Nettle Additive on Liver Enzyme Activity in Fish

Any change in the physiological status of the fish, from pollution to nutritional stress, can cause changes in liver enzymes. The fish liver is a key organ of numerous vital metabolic reactions related to carbohydrates, lipids, and protein [70]. Evaluation of liver indicators, including ALT, AST, lactate dehydrogenase (LDH), and ALP, provides a suitable index for the health condition of fish [71]. There is a long list of herbs that decrease these enzymes and eventually provide a healthier status (with a higher immunity and antioxidant response) for fish [72, 73]. Interestingly, in some of these investigations, fish with a lower level of liver enzymes also had a higher growth rate [33, 72, 74–80]. Studies regarding the effect of nettle on these enzymes are limited. Researchers found that nettle sourced from aerial parts with ethanol extract at levels 2% and 3% in rainbow trout and beluga did not significantly demonstrate an effect on blood serum ALT, AST, and LDH [33, 35]. It can be hypothesized that the levels used in these studies were not high enough (antinutritional factors are high as well) to affect fish negatively. Usually, if the herbs are high in fish diets, liver enzymes tend to be higher to reduce or eliminate antinutritional factors from the fish body. However, more studies are required to test this hypothesis. The effects of supplementation nettle on liver enzyme activity in fish are displayed in Table 4.

## 7. Effects of Nettle on the Immune System of Fish

An immunostimulant is a substance, medicine, or compound that improves the defensive system and immune response of fish [81]. Herbal medicine has been considered a strong immunostimulant, and numerous herbs have indicated this effect in aquaculture species [6, 82]. Generally, the most studied positive effects of herbal medicine in fish are related to its immunostimulatory effect. Several studies have indicated the positive impact of nettle in fish. This effect is due to bioactive compounds, including phenolic compounds and flavonoids [27].

We observed that 8 weeks of feeding rainbow trout (7 g) with a diet content of 3% nettle leaf powder improved blood serum lysozyme enzyme activity [22]. Moreover, others found that supplementation of this herb in rainbow trout at levels 0.5, 1, and 1.5% after 4 weeks of feeding did not improve respiratory burst activity and blood serum alternative complement activity (ACH50), but in the 0.5% group, the serum lysozyme was higher than in the controls [37]. In another study in this fish species (10 g), after 8 weeks of feeding nettle leaf powder at dietary levels of 0.5%, 1%, and 1.5%, increased respiratory burst activity and blood serum lysozyme enzyme ACH50 were observed [24]. Moreover, Awad and Austin [21] reported that supplementing 1% nettle leaf powder to the diet of rainbow trout (15 g) improved respiratory burst, blood serum lysozyme, and bactericidal activity, and no change in phagocytic activity, blood serum *α*2-macroglobulin, myeloperoxidase content, complement, or antiprotease activity after 14 days feeding was observed. After 30 days of providing rainbow trout (10 g) with nettle methanol extract at dietary levels of 0.01% and 0.05%, increased phagocytic activity, blood serum lysozyme, and myeloperoxidase activity were reported [40].

Use of 0.01% and 0.05% methanolic extracts of nettle in female convict cichlid diet significantly increased complement component 3, complement component 4, and IgM levels at a level of 0.01% of the diet compared to the control [43]. In this context, Ngugi et al. [36] reported that nettle leaf powder in Victoria Labeo juvenile and adult diets with an initial average body weight of 25 g at levels 1%, 2%, and 5% stimulated respiratory burst and blood serum lysozyme activity significantly after 4- and 16-week feeding trials. Another study showed that nettle leaf powder at dietary levels of 3%, 6%, and 12% in beluga (11 g) did not stimulate the immune system, including blood serum total immunoglobulin level, lysozyme, and respiratory burst activity after a 4-week feeding trial [35]. By contrast, after 8 weeks of feeding, total blood serum immunoglobulin levels and respiratory burst activity significantly increased in beluga (204 g) in the 12% nettle group [35]. Improvement of nonspecific immune parameters can be interpreted as the effect of the nettle supplement in the diet of fish due to its different essential amino acids [47] and phenolic compounds, including quercetin, kaempferol, myricetin, and rutin [67], especially quercetin [23], which is known as a phenolic compound, and stimulated immune parameters in fish [21, 24, 33, 36, 37]. Nettle can boost serum total protein level and immune response of fish, as previously mentioned. The effects of supplementing nettle in the fish diet on the immune system are shown in Table 5.

## 8. Nettle Helps Fish Fight Pathogens

A long list of herbs has positively affected fishes' ability to fight against pathogens [80, 83]. Challenging fish with pathogens has been considered one of the most common and best ways to measure fish and larvae quality. Numerous parameters, from stress to nutritional change, can alter the stress response and eventually the survival rate of fish. Herbal medicine has been one of the greenest ways to improve fish resistance to pathogens. In the past few decades, nettle powder and/or extract have been used as feed additives in fish diets to increase immune resistance and combat pathogens [21, 24, 33, 40].

Mehrabi et al. [24] reported that supplementing 0.5% nettle leaf powder in rainbow trout for 8 weeks showed the highest survival rate (86%) after 3 weeks of challenge with *Saprolegnia parasitica* compared to the control with a 56% survival rate. Moreover, others reported that 1% nettle leaf powder in rainbow trout (15 g) after 14 days of feeding showed the highest survival rate (96%) compared to the control (32%) after 10 days of an *A. hydrophila* challenge [21]. Similarly, a higher survival rate in nettle-supplemented groups (50%-80%) compared with the 35% survival rate in the control in rainbow trout (42 g) after 8 weeks of feeding, followed by 14 days of a *Yersinia ruckeri* challenge, was observed [33]. Bilen et al. [40] used 0.01% and 0.05% nettle methanol extracts in a rainbow trout diet with an initial average body weight of 10 g after a 30-day feeding trial, and their results showed that nettle groups achieved the highest survival rate compared to the controls after a 14-day *A. hydrophila* challenge. In warm-water species, the same improvement in survival rate after a challenge by nettle was observed. Therefore, the use of 1%, 2%, and 5% nettle leaf powder in Victoria Labeo after 4 and 16 weeks of feeding could increase the survival rate (more than 80%) in nettle's groups (2% and 5%) compared to the control group (0%) after a 21-day *A. hydrophila* challenge [36].

There are many possible explanations behind this improvement by nettle. The antimicrobial activity of phenolic components of nettle, such as carvacrol, thymol, and quercetin, has been well documented [7, 84]. Moreover, quercetin has a wide range of biological functions that modulate oxidative stress and inflammatory reactions in mammals [85]. Administration of nettle powder and or extract in fish diet could improve WBC and stimulate nonspecific immune parameters, including immunoglobulin, lysozyme, respiratory burst, ACH50, phagocytic, complements, bactericidal, and antiprotease activity in fish, and increased resistance against pathogens [21, 24, 36, 40]. All these parameters can improve stress responsiveness and survival rates separately or with each other. Furthermore, in terms of gene expression levels, some evidence has indicated that nettle could have positive effects. Cytokines are small protein intermediaries created by immune cells that regulate the immune system, inflammation, and hematopoiesis [86]. Some relative cytokine genes, including interleukin 6 (IL-6) and interleukin 8 (IL-8), showed high expression in nettle treatments [24]. IL-6 is a key multifunctional cytokine that is known as a B-cell separation factor involved in the maturation of antibody-producing cells in mammals [87], and IL-6 is expressed at high levels in inflammatory diseases in mammals [88]. IL-8 is strongly associated with cytokines. It is created by phagocytes and mesenchymal cells exposed to inflammatory stimulants and activates neutrophils and respiratory bursts in fishes and mammals [21, 24, 33, 89]. Moreover, other genes, such as tumor necrosis factor *α* (TNF-*α*) and interleukin 1 beta (IL-1*β*), showed high expression in nettle treatments in fish [24]. TNF-*α* is one of the cytokines involved in developing inflammation and regulating immune cells in different animals and humans [90]. It removes pathogens during the induction of different cellular responses [91]. Furthermore, IL-1*β* has a central role in response to pathogens and tissue damage in various taxa [92], and it controls immune responses by increasing cytokines [93]. The effects of the application of nettle in fish diets in terms of ability to fight against pathogens are presented in Table 6.

## 9. Conclusion

Literature on the applications of nettle has been reviewed on various aquaculture species, with a focus on important and common aquaculture species such as rainbow trout. Nettle has useful features, including growth promotion, immune-boosting, antiviral, antibacterial, and antiparasitic activities. It also improved hematological and blood biochemistry and lowered stress. Nettle improved most of these features in different sizes or species of fish. Natural additives such as nettle could represent a green and sustainable replacement for hormones, antibiotics, and chemical drugs to enhance growth performance, the immune system, and resistance against pathogens due to active compounds, including alkaloids, flavonoids, phenolics, terpenoids, pigments, minerals, and essential oils. Nettle can potentially help to produce healthy food, increase aquaculture production quality and quantity, and reduce environmental pollution through the reduction of chemical usage in aquafeed and fish farm effluent. The combination of molecular studies on gene levels, proteins, and omics (i.e., transcriptomics, proteomics, metabolomics, and microbiomics) in addition to classic analysis can provide a better understanding of nettle's mechanism of action, as well as promote the use of this herb in aquaculture. Finally, reaching a plateau in growth and finding the optimum dosage is necessary, as various fish species have different requirements to achieve maximum growth.

## Figures and Tables

**Table 1:** Effects of nettle (*Urtica dioica*) supplement on growth performance and body proximate composition of fish.

Fish species	Nettle part used	Preparation of nettle supplement	Type of plant additive	Doses (%)	Period of feeding trial	Active compounds	Optimum dose	Beneficial effects on growth performance and body proximate composition in fish significantly	References
Rainbow trout (*Oncorhynchus mykiss*)	Leaves	Dried in shade at room temperature	Powder	1% and 3%	8 weeks	Phenolic compounds and amino acids	3%	FW, WG, WG%, SGR%, body proximate protein, fat, and ash	[16]
Rainbow trout	Leaves	Dried in shade	Powder	0.5, 1, 1.5%	8 weeks	Capsaicin, carvacrol, cinnamaldehyde	0.5%, 1%, and 1.5%	FW, WG, SGR%, FCR	[18]
Rainbow trout	Leaves	Dried in the shade under ambient conditions by a flow of dry air	Powder	0.5, 1, 1.5%	4 weeks	Acetylcholine, histamine, serotonin, salicylic acid, lecithin, carotenoid, flavonoids, sterols, and thymol	0.5, 1%, and 1.5%	FW, WG, SGR%, FCR, and PER	[31]
Rainbow trout	Leaves	Dried leaves from a healthy food shop in Edinburgh	Powder	1% and 2%	2 months	Amino acids	1% and 2%	FW, WG, and SGR%	[47]
Rainbow trout	Leaves	Dried leaves obtained from Good n' Natural, Nuneaton, UK	Powder	1%	14 days	Not reported	—	Growth performance and body proximate composition were not evaluated	[15]
Victoria Labeo (*Labeo victorianus*)	Leaves	Air-dried	Powder	1%, 2%, and 5%	16 weeks	Vitamin A, vitamin B, vitamin B12, acetylcholine, serotonin, formic acid, salicylic acid, lecithin, carotenoids, flavonoids, sterols, and thymol	1%, 2%, and 5%	FW, WG%, SGR%, and FCR	[30]
Beluga (*Huso huso*)	Leaves and stem	Not reported	Powder	3%, 6%, and 12%	8 weeks	Flavonoids, polysaccharides, carotenoids, lignans, lectins, amino acids, minerals (especially iron) and vitamins (vitamin C), tannins, formic acid, salicylic acid, carvacrol, and thymol	12%	Growth performance and body proximate composition were not evaluated	[29]
Beluga	Not reported	Oven-dried was obtained from the supermarket	Powder	1%	60 days	Acetylcholine, serotonin, formic acid, minerals, amino acid, lecithin, carotenoid, flavonoids, sterols, tannins, and vitamins	1%	Growth performance was not significant	[94]
Electric yellow cichlid (*Labidochromis caeruleus*)	Not reported	Dried under shadow	Extract	0.015%	3 months	Carotenoids	0.015%	Growth performance was not significant	[32]
Convict cichlid (*Cichlasoma nigrofasciatum*)	Root	Ethanol extract	Extract	0.02% and 0.03%	8 weeks	Flavonoids	—	Reduced FW, WG%, and SGR%	[33]
Rainbow trout	Aerial parts	Ethanol extract	Extract	1%, 2%, and 3%	8 weeks	Carvacrol, flavonoids, carotenoids proteins, iron, and vitamins	3%	WG, SGR%, FCR, and SR%	[28]
Rainbow trout	Not reported	Methanol extract	Extract	0.01% and 0.05%	30 days	Quercetin	0.01% and 0.5%	FW, WG, SGR%, and FCR	[34]
Rainbow trout	Not reported	Nettle extract (quercetin) obtained from the laboratory of professor Amani Awaad	Extract	0.1%, 0.5%, and 1%	14 days	Quercetin	1%	Growth performance and body proximate composition were not evaluated	[17]
Convict cichlid (*Amatitlania nigrofasciata*)	Leaves	Methanol extract	Extract	0.01% and 0.05%	56 days	Capsaicin and carvacrol	0.01%	WG, SGR, FI, FCR, and PER	[48]

**Table 2:** Effects of nettle supplement on hematological parameters of fish.

Fish species	Nettle part used	Preparation of nettle supplement	Type of plant additive	Doses (%)	Period of feeding trial	Active compounds	Optimum dose	Beneficial effects on hematological parameters of fish significantly	References
Rainbow trout	Leaves	Dried in shade at room temperature	Powder	1% and 3%	8 weeks	Phenolic compounds and amino acids	1% and 3%	WBC, RBC, Hct, Hb, monocyte, and lymphocyte	[16]
Rainbow trout	Leaves	Dried in shade	Powder	0.5, 1, 1.5%	8 weeks	Capsaicin, carvacrol, cinnamaldehyde	0.5%, 1%, and 1.5%	WBC, RBC, Hct, Hb, and lymphocyte	[18]
Rainbow trout	Leaves	Dried in the shade under ambient condition by a flow of dry air	Powder	0.5%, 1%, 1.5%	4 weeks	Acetylcholine, histamine, serotonin, salicylic acid, lecithin, carotenoid, flavonoids, sterols, and thymol	0.5%, 1%, and 1.5%	WBC, RBC, Hct, and Hb	[31]
Rainbow trout	Leaves	Dried leaves from a health food shop in Edinburgh	Powder	1% and 2%	2 months	Amino acids	1% and 2%	Hematology was not evaluated	[47]
Rainbow trout	Leaves	Dried leaves obtained from Good n' Natural, Nuneaton, UK	Powder	1%	14 days	Not reported	1%	Hb	[15]
Victoria Labeo	Leaves	Air-dried	Powder	1%, 2%, and 5%	16 weeks	Vitamin A, vitamin B, vitamin B12, acetylcholine, serotonin, formic acid, salicylic acid, lecithin, carotenoids, flavonoids, sterols, and thymol	1%, 2%, and 5%	WBC, RBC, Hct, Hb, and neutrophil	[30]
Beluga	Leaves and stem	Not reported	Powder	3%, 6%, and 12%	8 weeks	Flavonoids, polysaccharides, carotenoids, lignans, lectins, amino acids, minerals (especially iron) and vitamins (vitamin C), tannins, formic acid, salicylic acid, carvacrol, and thymol	12%	WBC, RBC, Hct, Hb, and neutrophil	[29]
Beluga	Not reported	Oven-dried was obtained from supermarket	Powder	1%	60 days	Acetylcholine, serotonin, formic acid, minerals, amino acid, lecithin, carotenoid, flavonoids, sterols, tannins, and vitamins	1%	Hct	[94]
Electric yellow cichlid	Not reported	Dried under shadow	Extract	0.015%	3 months	Carotenoids	0.015%	Hematology was not evaluated	[32]
Convict cichlid	Root	Ethanol extract	Extract	0.02% and 0.03%	8 weeks	Flavonoids	—	Hematology was not evaluated	[33]
Rainbow trout	Aerial parts	Ethanol extract	Extract	1%, 2%, and 3%	8 weeks	Carvacrol, flavonoids, carotenoids proteins, iron, and vitamins	3%	WBC, RBC, Ht, Hb, lymphocyte, and neutrophil	[28]
Rainbow trout	Not reported	Methanol extract	Extract	0.01% and 0.05%	30 days	Quercetin	0.01% and 0.5%	Hematology was not evaluated	[34]
Rainbow trout	Not reported	Nettle extract (quercetin) obtained from the laboratory of professor Amani Awaad	Extract	0.1%, 0.5%, and 1%	14 days	Quercetin	1%	Hematology was not evaluated	[17]
Convict cichlid	Leaves	Methanol extract	Extract	0.01% and 0.05%	56 days	—	—	Hematology was not evaluated	[48]

**Table 3:** Effects of nettle supplement on blood biochemical parameters of fish.

Fish species	Nettle (*U. dioica*) part used	Preparation of nettle (*U. dioica*) supplement	Type of plant additive	Doses (%)	Period of feeding trial	Active compounds	Optimum dose	Beneficial effects on blood biochemical parameters on fish significantly	References
Rainbow trout	Leaves	Dried in shade at room temperature	Powder	1% and 3%	8 weeks	Phenolic compounds and amino acids	1% and 3%	Albumin and total protein	[16]
Rainbow trout	Leaves	Dried in shade	Powder	0.5, 1, 1.5%	8 weeks	Capsaicin, carvacrol, cinnamaldehyde	0.5%, 1%, and 1.5%	Albumin, total protein, and globulin	[18]
Rainbow trout	Leaves	Dried in the shade under ambient condition by a flow of dry air	Powder	0.5, 1, 1.5%	4 weeks	Acetylcholine, histamine, serotonin, salicylic acid, lecithin, carotenoid, flavonoids, sterols, and thymol	0.5%, 1%, and 1.5%	Albumin, total protein, and globulin	[31]
Rainbow trout	Leaves	Dried leaves from a health food shop in Edinburgh	Powder	1% and 2%	2 months	Amino acids	—	Blood biochemistry was not evaluated	[47]
Rainbow trout	Leaves	Dried leaves obtained from Good n' Natural, Nuneaton, UK	Powder	1%	14 days	Not reported	1%	No significant effect	[15]
Victoria Labeo	Leaves	Air-dried	Powder	1%, 2%, and 5%	16 weeks	Vitamin A, vitamin B, vitamin B12, acetylcholine, serotonin, formic acid, salicylic acid, lecithin, carotenoid, flavonoids, sterols, and thymol	1%, 2%, and 5%	Albumin, total protein, triglyceride, cholesterol, cortisol, and glucose	[30]
Beluga	Leaves and stem	Not reported	Powder	3%, 6%, and 12%	8 weeks	Flavonoids, polysaccharides, carotenoids, lignans, lectins, amino acids, minerals (especially iron) and vitamins (vitamin C), tannins, formic acid, salicylic acid, carvacrol, and thymol	12%	Total protein, triglyceride, and cholesterol	[29]
Beluga	Not reported	Oven-dried was obtained from supermarket	Powder	1%	60 days	Acetylcholine, serotonin, formic acid, minerals, amino acid, lecithin, carotenoid, flavonoids, sterols, tannins, and vitamins	—	Blood biochemistry was not evaluated	[94]
Electric yellow cichlid	Not reported	Dried under shadow	Extract	0.015%	3 months	Carotenoids	—	Blood biochemistry was not evaluated	[32]
Convict cichlid	Root	Ethanol extract	Extract	0.02% and 0.03%	8 weeks	Flavonoids	—	Blood biochemistry was not evaluated	[33]
Rainbow trout	Aerial parts	Ethanol extract	Extract	1%, 2%, and 3%	8 weeks	Carvacrol, flavonoids, carotenoids proteins, iron, and vitamins	3%	Total protein and triglyceride	[28]
Rainbow trout	Not reported	Methanol extract	Extract	0.01% and 0.05%	30 days	Quercetin	—	Blood biochemistry was not evaluated	[34]
Rainbow trout	Not reported	Nettle extract (quercetin) obtained from the laboratory of professor Amani Awaad	Extract	0.1%, 0.5%, and 1%	14 days	Quercetin	1%	Total protein	[17]
Convict cichlid	Leaves	Methanol extract	Extract	0.01% and 0.05%	56 days	Carvacrol	0.01%	Glucose, triglyceride, cholesterol, total protein, and albumin	[48]

**Table 4:** Effects of nettle supplement in fish liver enzyme activity.

Fish species	Nettle part used	Preparation of nettle supplement	Type of plant additive	Doses (%)	Period of feeding trial	Active compounds	Optimum dose	Measured liver enzymes in blood serum	Significant effect	References
Beluga	Leaves and stem	Not reported	Powder	3%, 6%, and 12%	8 weeks	Flavonoids, polysaccharides, carotenoids, lignans, lectins, amino acids, minerals (especially iron) and vitamins (vitamin C), tannins, formic acid, salicylic acid, carvacrol, and thymol	—	ALT, AST, and ALP	No significant	[29]
Rainbow trout	Aerial parts	Ethanol extract	Extract	1%, 2%, and 3%	8 weeks	Carvacrol, flavonoids, carotenoids proteins, iron, and vitamins	—	ALT, AST, and LDH	No significant	[28]

ALT: alanine aminotransferase; AST: aspartate aminotransferase; ALP: alkaline phosphatase; LDH: lactate dehydrogenase.

**Table 5:** Effects of nettle supplement on immunological parameters of fish.

Fish species	Nettle part used	Preparation of nettle supplement	Type of plant additive	Doses (%)	Period of feeding trial	Active compounds	Optimum dose	Beneficial effects of immunological parameters of fish significantly	References
Rainbow trout	Leaves	Dried in shade at room temperature	Powder	1% and 3%	8 weeks	Phenolic compounds and amino acids	1% and 3%	Lysozyme activity and immunoglobulin	[16]
Rainbow trout	Leaves	Dried in shade	Powder	0.5%, 1%, and 1.5%	8 weeks	Capsaicin, carvacrol, cinnamaldehyde	0.5%, 1%, and 1.5%	Respiratory burst activity, lysozyme activity, and ACH50	[18]
Rainbow trout	Leaves	Dried in the shade under ambient condition by a flow of dry air	Powder	0.5%, 1%, and 1.5%	4 weeks	Acetylcholine, histamine, serotonin, salicylic acid, lecithin, carotenoid, flavonoids, sterols, and thymol	0.5%	Lysozyme activity	[31]
Rainbow trout	Leaves	Dried leaves from a health food shop in Edinburgh	Powder	1% and 2%	2 months	Amino acids	—	Immunology was not evaluated	[47]
Rainbow trout	Leaves	Dried leaves obtained from Good n' Natural, Nuneaton, UK	Powder	1%	14 days	Not reported	1%	Lysozyme, respiratory burst, and bactericidal activity	[15]
Victoria Labeo	Leaves	Air-dried	Powder	1%, 2%, and 5%	16 weeks	Vitamin A, vitamin B, vitamin B12, acetylcholine, serotonin, formic acid, salicylic acid, lecithin, carotenoid, flavonoids, sterols, and thymol	1%, 2%, and 5%	Total immunoglobulin (Ig), respiratory burst, and lysozyme activity	[30]
Beluga	Leaves and stem	Not reported	Powder	3%, 6%, and 12%	8 weeks	Flavonoids, polysaccharides, carotenoids, lignans, lectins, amino acids, minerals (especially iron) and vitamins (vitamin C), tannins, formic acid, salicylic acid, carvacrol, and thymol	12%	Total immunoglobulin (Ig) and respiratory burst activity	[29]
Beluga	Not reported	Oven-dried was obtained from supermarket	Powder	1%	60 days	Acetylcholine, serotonin, formic acid, minerals, amino acid, lecithin, carotenoid, flavonoids, sterols, tannins, and vitamins	1%	Immunology was not evaluated	[94]
Electric yellow cichlid	Not reported	Dried under shadow	Extract	0.015%	3 months	Carotenoids	0.015%	Immunology was not evaluated	[32]
Convict cichlid	Root	Ethanol extract	Extract	0.02% and 0.03%	8 weeks	Flavonoids	—	Immunology was not evaluated	[33]
Rainbow trout	Aerial parts	Ethanol extract	Extract	1, 2, and 3%	8 weeks	Carvacrol, flavonoids, carotenoids proteins, iron, and vitamins	3%	Immunoglobulin (IgM), complement 3, complement 4, lysozyme, and respiratory burst activity	[28]
Rainbow trout	Not reported	Methanol extract	Extract	0.01% and 0.05%	30 days	Quercetin	0.01% and 0.05%	Phagocytic, lysozyme, and myeloperoxidase activity	[34]
Rainbow trout	Not reported	Nettle extract (quercetin) obtained from the laboratory of professor Amani Awaad	Extract	0.1%, 0.5%, and 1%	14 days	Quercetin	0.5% and 1%	Lysozyme, myeloperoxidase, bactericidal, antiprotease activity, and immunoglobulin (IgM)	[17]
Convict cichlid	Leaves	Methanol extract	Extract	0.01% and 0.05%	56 days	Quercetin	0.01%	C3, C4, and IgM	[48]

**Table 6:** Effects of nettle supplement on pathogen challenge and survival rate in fish.

Fish species	Nettle part used	Preparation of nettle supplement	Type of plant additive	Doses (%)	Period of feeding trial	Active compounds	Optimum dose	Pathogen species	Pathogen challenge period	Survival rate % in nettle groups after challenge significantly	Survival rate % in control after challenge	References
Rainbow trout	Leaves	Dried in shade at room temperature	Powder	1% and 3%	8 weeks	Phenolic compounds and amino acids	—	—	—	—		[16]
Rainbow trout	Leaves	Dried in shade	Powder	0.5, 1, 1.5%	8 weeks	Capsaicin, carvacrol, cinnamaldehyde	0.5%	*Saprolegnia parasitica*	3 weeks	86%	56%	[18]
Rainbow trout	Leaves	Dried in the shade under ambient condition by a flow of dry air	Powder	0.5%, 1%, and 1.5%	4 weeks	Acetylcholine, histamine, serotonin, salicylic acid, lecithin, carotenoid, flavonoids, sterols, and thymol	—	—	—	—	—	[31]
Rainbow trout	Leaves	Dried leaves from a healthy food Shop in Edinburgh	Powder	1% and 2%	2 months	Amino acids	—	—	—	—	—	[47]
Rainbow trout	Leaves	Dried leaves obtained from Good n' Natural, Nuneaton, UK	Powder	1%	14 days	Not reported	1%	*Aeromonas hydrophila*	10 days	96%	32%	[15]
Victoria Labeo	Leaves	Air-dried	Powder	1%, 2%, and 5%	16 weeks	Vitamin A, vitamin B, vitamin B12, acetylcholine, serotonin, formic acid, salicylic acid, lecithin, carotenoid, flavonoids, sterols, and thymol	1, 2, and 5%	*Aeromonas hydrophila*	21 days	50, 88, and 96%, respectively	0%	[30]
Beluga	Leaves and stem	Not reported	Powder	3%, 6%, and 12%	8 weeks	Flavonoids, polysaccharides, carotenoids, lignans, lectins, amino acids, minerals (especially iron) and vitamins (vitamin C), tannins, formic acid, salicylic acid, carvacrol, and thymol	—	—	—	—	—	[29]
Beluga	Not reported	Oven-dried was obtained from the supermarket	Powder	1%	60 days	Acetylcholine, serotonin, formic acid, minerals, amino acid, lecithin, carotenoid, flavonoids, sterols, tannins, and vitamins	—	—	—	—	—	[94]
Electric yellow cichlid	Not reported	Dried under shadow	Extract	0.015%	3 months	Carotenoids	—	—	—	—	—	[32]
Convict cichlid	Root	Ethanol extract	Extract	0.02% and 0.03%	8 weeks	Flavonoids	—	—	—	—	—	[33]
Rainbow trout	Aerial parts	Ethanol extract	Extract	1%, 2%, and 3%	8 weeks	Carvacrol, flavonoids, carotenoids proteins, iron, and vitamins	3%	*Yersinia ruckeri*	14 days	78%	41%	[28]
Rainbow trout	Not reported	Methanol extract	Extract	0.01% and 0.05%	30 days	Quercetin	0.01% and 0.05%	*Aeromonas hydrophila*	14 days	58%	0%	[34]
Rainbow trout	Not reported	Nettle extract (quercetin) obtained from the laboratory of professor Amani Awaad	Extract	0.1%, 0.5%, and 1%	14 days	Quercetin	—	*—*	—	—	—	[17]

## Supplementary Materials

Supplementary Materials The supplementary file 2 presents different isolated compounds, type of compounds, and level of compounds from aerial parts, leaves, stems, flowers, seeds, and roots of nettle.
