# Supplementary material for: Nettle (Urtica dioica) Additive as a Growth Promoter and Immune Stimulator in Fish
Source: Aquac Nutr. 2023 Feb 21;2023:8261473. doi: 10.1155/2023/8261473 (PMC9974277; doi:10.1155/2023/8261473)
Supplement: Supplementary Materials — The supplementary file 2 presents different isolated compounds, type of compounds, and level of compounds from aerial parts, leaves, stems, flowers, seeds, and roots of nettle. [file 8261473.f1.docx]

| Supplementary file 2. The compounds were isolated from aerial parts, leaves, stems, flowers, seeds, and roots of nettle | | | | |
| --- | --- | --- | --- | --- |
| Compounds | Type of compounds | Level of compounds | Nettle part | References |
| Essential amino acids | Alanine  4-aminobutyrate (GABA)  Glutamic acid  Isoleucine  Leucine  Phenylalanine  Proline  Tyrosine  Valine | 6.4 µg/g  3.4 µg/g  6.0 µg/g  1.9 µg/g  7.6 µg/g  2.4 µg/g  5.7 µg/g  1.2 µg/g  3.0 µg/g | Leaves | [1] |
| Organic acids | Malic acid  Acetic acid  Citric acid  Succinic acid  Formic acid | 12.8 µg/g  29.3 µg/g  3.6 µg/g  2.6 µg/g  6.9 µg/g | Leaves | [1] |
| Lipids | steroids and triterpenoids 3-b-sitosterol  sitosterol-b-D-glucoside  (60 -O-palmitoyl)-sitosterol-3- O-b-D-glucoside  24R-ethyl-5a-cholestane3b,6a-diol  7b-hydroxy-sitosterol  7a-hydroxy sitosterol  7b-hydroxy-sitosterol-b-Dglucoside  7a-hydroxy-sitosterol-b-Dglucoside | 40 mg/kg  30 mg/kg  5.6 mg/kg  3.3 mg/kg  2.4 mg/kg  2.9 mg/kg  2.7 mg/kg  2.0 mg/kg | Root | [1] |
| Fatty acids | terpenoids, phenolic compounds, and volatile compounds | - | Leaves, roots, seeds, and stems | [1] |
| Fat-soluble vitamins | A  D  E  K | -  -  14.4 (mg/100g)  - | Aerial parts, leaves, stems, flowers, seeds, and roots | [2] |
| Water-soluble vitamins | B1  B2 (riboflavin)  B3 (Niacin)  B6  C  A (β-carotene) | 0.01 (mg/100g)  0.23 (mg/100g)  0.26 (mg/100g)  0.068 (mg/100g)  238 (mg/100g)  5 (mg/100g) | leaves | [2] |
| Minerals | Calcium  Magnesium  Phosphorus  Potassium  Sodium  Cobalt  Copper  Iron  Selenium  Zinc  Manganese | (113.2 –5,090 mg/100 g of dry matter)  (0.22 –3,560 mg/100 g)  (29 –75 mg/100 g)  (532–917.2 mg/100 g)  (5.5–16 mg/100 g)  (0.0084 –0.018 mg/100 g)  (0.52 –1.747 mg/100 g)  (3.4 –30.30 mg/100 g)  (0.0027 –0.0047 mg/100 g),  (0.9 –3.033 mg/100 g)  (0.768 –5.784 mg/100 g) | Leaves | [3] |

**References**

1. L. Grauso, B. de Falco, V. Lanzotti and R. Motti, "Stinging nettle, (*Urtica dioica* L.): Botanical, phytochemical and pharmacological overview," *Phytochemistry Reviews*, vol. 19, pp. 1341-1377, 2020.

2. P. Bagade, V. Pant and S. T. Pandey, "Nutritional and antinutritional profiling of stinging nettle (*Urtica dioica* L.)," *International Journal of Food Science and Nutrition*, vol. 6, no. 4, pp. 111-116, 2021.

3. A. A. H. Said, I. Otmani, S. Derfoufi and A. Benmoussa, "Highlights on nutritional and therapeutic value of stinging nettle (*Urtica dioica*)," *International Journal of Pharmacy and Pharmaceutical Sciences*, vol. 7, no. 10, pp. 8-14, 2015.
